# Study on a Flexible Odor-Releasing Device for Olfactory Training

**DOI:** 10.3390/s22239519

**Published:** 2022-12-06

**Authors:** Huisheng Peng, Cheng Yang, Feitong Jian, Shuo Wu

**Affiliations:** 1 School of Aeronautics and Astronautics, Sun Yat-sen University, Guangzhou 510275, China; 2 Department of Otorhinolaryngology Head and Neck Surgery, The Third Affiliated Hospital of Sun Yat-sen University, Guangzhou 510630, China

**Keywords:** olfactory training, olfactory dysfunction, odor generation, ultrasonic atomizer, odorant, 3D printing

## Abstract

Olfactory training has been shown to be effective in treating olfactory dysfunction. However, there are hardly any devices that can regularly and quantificationally release odors for olfactory training. A new odor-releasing device, which is low-cost, customizable, semi-automatic, and flexible, was developed in this study. The operation of the device can be easily achieved by the examiner, or even by the participant, simply by pressing a few buttons. A controller system with 15 individual relays was employed to master the working logic for the whole process. The device allows the examiner to isolate from the participants using the Bluetooth module in the control board. The odorants and their concentrations stored in the scent bottles can be customized by the specific requirements of different participants. The odors for training are provided by ultrasonic atomizers, which have simple structures, but powerful features. The flow rates of the odors can also be controlled by altering the rotation speed of the fans. Final experiments on practical odor generation further proved the potential of the developed device for olfactory training. More attention should be paid to the improvements of odor generation devices for olfactory training.

## 1. Introduction

The olfactory sensation is one of the essential methods for humans to perceive the world. Based on different smells, we can distinguish the quality of different items, estimate the safety of environments, and even recall memories. Without it, the safety and quality of our lives would be severely affected. Currently, many people are losing their olfactory sensation for various reasons (e.g., COVID-19, injury, and chronic sinusitis) [1]. Helping them recover from the dysfunction it is significant task. Studies have proved that damaged olfactory nerve cells have the ability to regenerate, proving the possibility of restoring olfactory sensation [2]. A method called olfactory training was recently proposed and used to treat this dysfunction, and this method has received wide recognition [3,4,5,6]. Olfactory training is a non-medical treatment wherein the participants conduct daily quantitative sniffing training using different smells for a certain period of time. By regular exposure to the odorant, the olfactory nerve can be activated and regenerated, gradually resulting in the recovery of the olfactory sensation.

In a common method of olfactory training, four odors—containing phenethyl alcohol, eucalyptol, citronellal, and eugenol—are employed. The participants are required to proactively smell each odorant for about 10 s twice daily for 12 weeks [7]. The clinical efficacy of this method has been validated, in many cases [7,8,9,10]. Researchers have also proposed some modified training methods by changing the odorants and their concentrations [11,12,13,14]; the results showed that the efficacy can be improved by extending the training time, widening the odor variety, and increasing the concentration. In addition, the brand or even the category of the odors can be randomly selected to achieve the same treatment efficacy [15], which can reduce the cost of the treatment. Since the efficacy of the olfactory training highly depends on the odorants, the devices storing the odorants are especially critical, which should accurately deliver the required odors to the participants during the training period.

**Figure 1:**
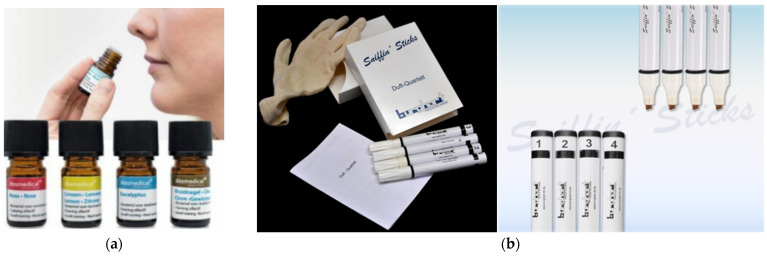
Display of the devices for olfactory training: (**a**) Dosmedical; (**b**) Sniffin’ Sticks.

For now, the sniffing bottles and sniffing sticks are the most widely used devices for olfactory training, which are based on the bottle (Figure 1a) and pen-like (Figure 1b) odor-dispensing devices, respectively. As shown, there are only four kinds of odors in the two kits. Each bottle (Figure 1a) contains 50 mL of odorant liquid or substance. For the training, the participants are required to sniff each bottle or stick for 3 to 4 s. Each bottle or stick must be used only once for each training. The sniffing time and the interval should be carefully controlled by the participants. To eliminate uncontrollable factors, the participants are forbidden to touch the devices, and the examiner must not give any hints to the participants. In addition, the training based on the two devices is required to be performed in a quiet, well-ventilated room, and both the participants and the examiners should be clean and odorless [16]. Obviously, olfactory training based on such devices is very complicated and tedious. The final treatment efficacy can be easily affected by many subjective factors, such as those mentioned above. In addition, these bottles or sticks will be opened and closed many times by the examiner and sniffed by different participants. Thus, the odorants in the sticks may be polluted by incautious handling after constant testing, which will gradually weaken the efficacy. The odor intake is also difficult to quantify regarding the free diffusion of the odors. Therefore, further studies should be conducted to improve this training method. It is urgent to develop a modified device to help better carry out the olfactory training.

In order to improve both the convenience and the accuracy of the olfactory training, in this study, a flexible semi-automatic device was developed for the training and built by 3D printing. Based on the device, the examiner only needs to press some buttons, and the device can accurately provide different odors, of different concentrations, to the participants. The values for overall training time, sniffing time, and the intervals between each subtest can be predefined by the device, replacing the manual controls in the sniffing sticks. Then, the device can automatically operate based on these settings, and the examiner only needs to wait for the end of each training. The participants can even conduct the training by themselves using the instructions regarding the device, avoiding the influence of the examiner. Moreover, the hardware for this device is all made up of cheap open components, which usually cost as little as several USD. The overall cost is much lower than that of than the sniffing sticks or dosages, making the treatment affordable for more participants. Details on this developed device are presented as follows.

The paper is organization as follows: this paper begins with an introduction to olfactory training and a review of the current methods (Section 1). Then, a novel device for olfactory training was developed, including the framework, odor generator, and controller, and the method is presented in Section 2. Section 3 shows the experimental results regarding the developed device to help assess its performance. Finally, the main findings and advice for future works are summarized in Section 4.

## 2. The Novel Device

The novel device developed in this study consists mainly of three parts: a framework, an odor generator, and a control system, with power supplies. Details of each are individually demonstrated in the following sections.

### 2.1. Framework

**Figure 2:**
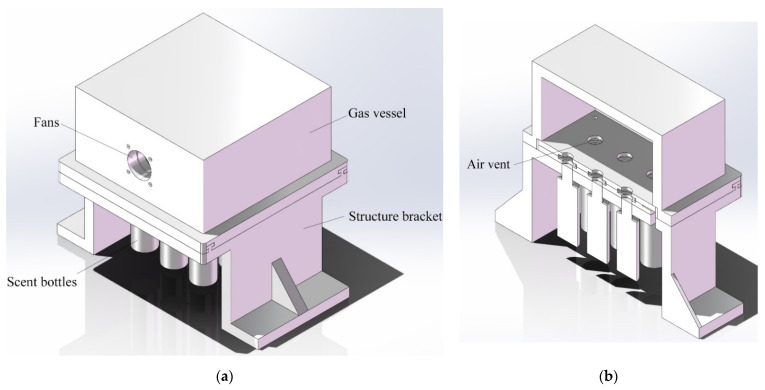
Scheme of the device framework: (**a**) isometric view; (**b**) section view.

Figure 2 shows the model diagram of the developed device framework. The overall structure can be divided into two parts: the gas vessel in the upper section, and the structure bracket in the bottom section; they are linked by two T-shaped connections, ensuring air-tightness between the two parts. The structural bracket is responsible for the stability of the device and supports the scent bottles and the gas vessel. A double L-shaped array geometry, with two ribs, was designed for better support and light weight. The gas vessel is a space for the storage and mixing of different odors released from the scent bottles, and they can be transported to the participants via fresh air using two fans. Different odorants with different concentrations are stored in nine bottles. These bottles are detachable, so the odorants can be conveniently replaced according to the different requirements of the participants, who may have different levels of olfactory dysfunction. To use the odorants, the odors will be released from the bottle by an odor generator and leaked to the gas vessel through the air vent above the bottles. These odors can be temporarily stored or mixed with other odors in the vessel, based on the command of the examiner.

**Figure 3:**
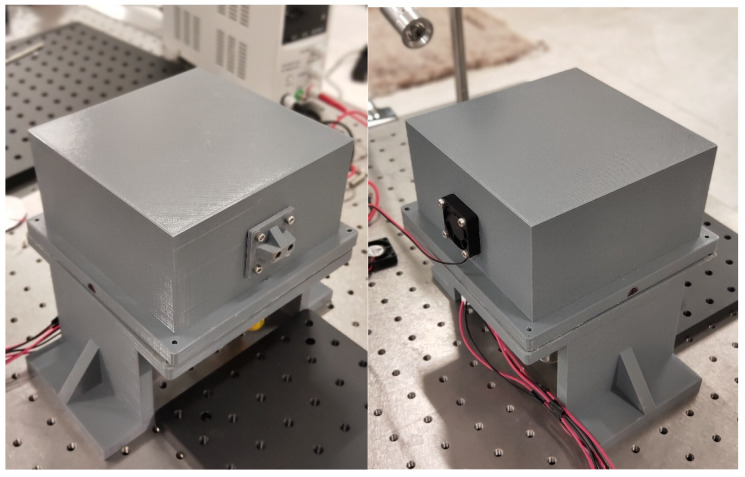
Illustration of the printed framework.

The overall measurements of the framework are 160 × 160 × 200 mm, which is about the size of the common sniffing sticks. It was constructed by 3D printing using PLA (polylactic acid) material. Figure 3 shows the illustration of the printed framework. Two fans were fixed on both sides of the gas vessel to blow out the odors contained in the vessel. In detail, the fan in the front panel absorbs the fresh air from the outside as the carrier gas. Then, the air mixes with the odors in the vessel. Finally, the fan inside the back panel exhausts the mixture from the vessel to the nose of the participant. Both the rotational speed and direction of the fans are adjustable to help reveal the relationships between the training effects and airflow velocity and flux. After each subtest, the fans can also help expel the odors that remaining in the vessel so as not to affect the next subtest. In addition, the thickness of the framework is less than 0.5 mm for light weight, and it can withstand the overall weight of all the components contained in the device. The overall printing cost of the framework is no more than USD 50, which is much lower than that of the sniffing sticks (USD 100+).

### 2.2. Odor Generator

**Figure 4:**
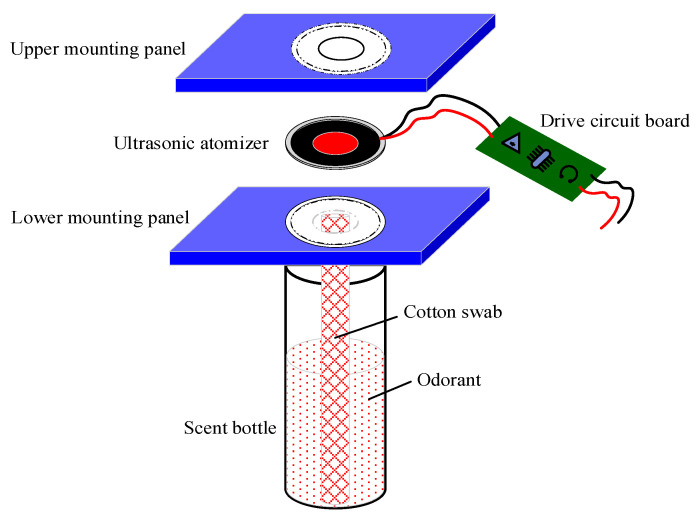
The overall scheme of the odor generator.

The odor generator, which is responsible for odor release, is the most essential part of the novel device. The generator consists of nine scent bottles, nine ultrasonic atomizers, two mounting panels, and several connecting wires. Figure 4 illustrates the overall scheme of the developed odor generator.

**Figure 5:**
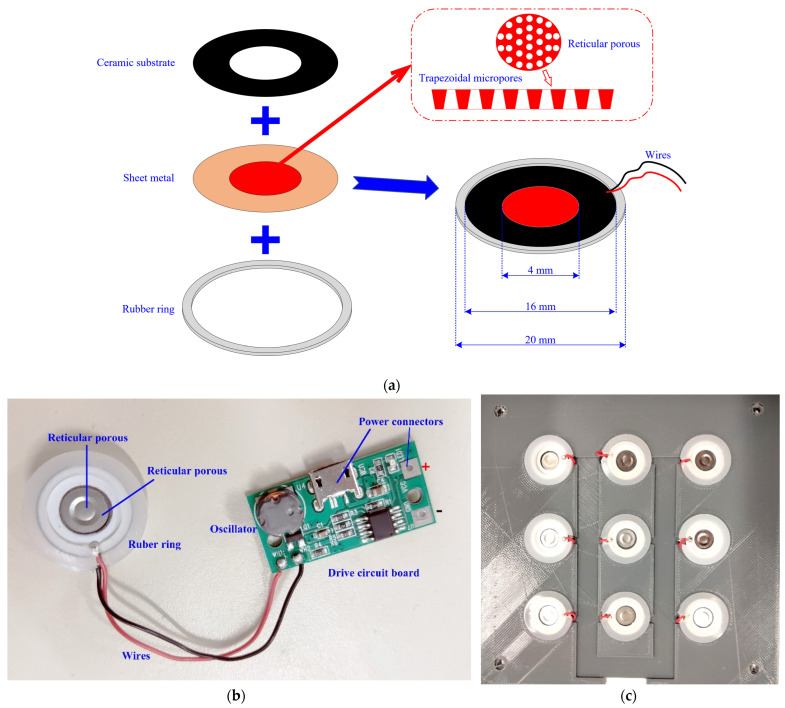
Scheme of the ultrasonic atomizer: (**a**) schematic diagram; (**b**) physical image; (**c**) assembly drawing.

In this study, the ultrasonic atomizer (Figure 5) was selected as the odor generator, which works based on the piezoelectric effect. In detail, the drive circuit (5 V) can generate a vibration wave of a certain frequency (108 kHz) and then convey it to the piezoelectric substrate. Owing to the piezoelectric effect, the vibration of the substrate can actuate the adjacent sheet metal to vibrate as well. The sheet exhibits a reticular porous structure containing hundreds of trapezoidal micropores. By the energy of their vibration, odorant liquids under the sheet can overcome the surface tension and be broken up into odor particles in the micropores. Then, these odor particles will be further diffused into the gas vessel through the air vent in the mounting panels. In addition, when the atomizer is not working, it can be used as a sealed cover over the scent bottles, both preventing the odors from leaking out and protecting them from pollution. The atomizer is also coated with a rubber ring for tightness in the junction. The overall size of the atomizer is 20 mm in diameter and 4 mm thick. Operations of the nine ultrasonic atomizers are then conducted. They do not impact each other. They can be switched on together or separately, based on the specific training requirements of the users, to provide single or mixed odors.

**Figure 6:**
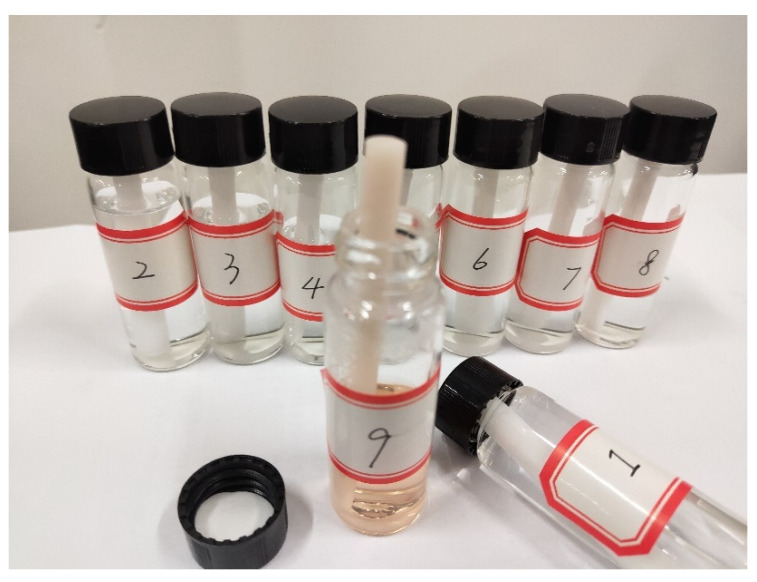
Scent bottles with a cotton swabs.

Different odorants are stored in nine transparent glass bottles (Figure 6), compared with the pen-like sticks used in the sniffing sticks. These bottles are commonly used reagent bottles and can hold 15 mL liquid odorants, sufficient for a full training dose. More importantly, they can be replenished several times, whereas the sticks are disposable. Users can conveniently monitor the odorant surplus through a transparent bottle. These bottles are screwed onto a mounting panel in the framework, and they can be conveniently changed through a screw thread when the liquids are used up. In addition, these glass bottles can be replaced by transparent plastic bottles for light weight. In order to make the atomizer fully accessible to the liquids, a 6 mm cotton swab was designed and placed in each bottle to link the liquid and the ultrasonic atomizer. The absorbency properties of cotton help infiltrate odorant liquids into the swab to reach the top side. Then, the liquids on the top will touch the ultrasonic atomizer and be atomized into odor particles.

**Figure 7:**
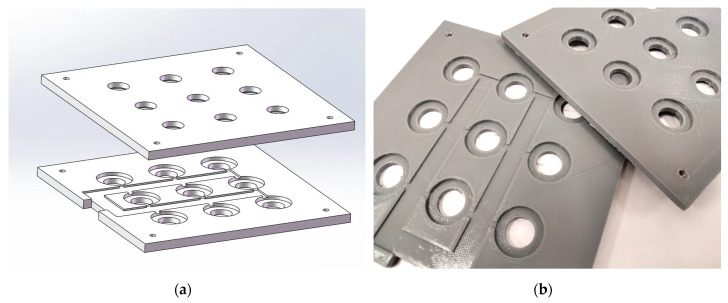
Mounting panels: (**a**) 3D design sketch; (**b**) printed entity.

Two mounting panels of 126 × 126 × 5 mm were designed for supporting and fixing of bottles and atomizers (Figure 7). On the bottom side of the lower panel, nine threaded holes (M18) are placed in the array of 3 × 3 for the installation of the scent bottles. On the opposite side, there are nine stepped holes of 20 × 2 mm for the placing of the atomizers. Moreover, two conduits are also designed on the lower panel for the wires, which are power cords for the atomizers. In order to match the lower panel, the upper panel also has nine stepped holes of 20 × 2 mm for the well-placing of atomizers on the bottom side. Nine through-holes of 12 mm are opened on the opposite side for the release of the odor particles from the atomizers into the gas vessel. These two panels are fixed on the corners by four nut-fastening bolts; they are also constructed by 3D printing, costing no more than USD 15.

### 2.3. Controller

**Figure 8:**
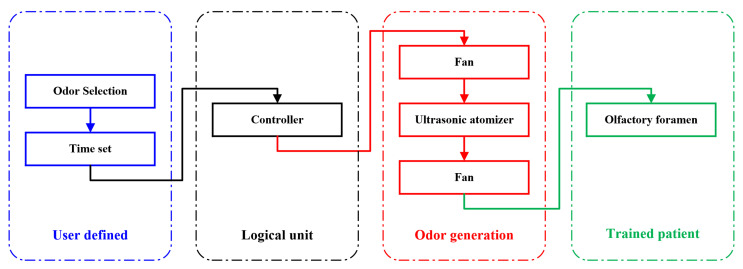
Workflow for olfactory training by the developed device.

Figure 8 illustrates the process workflow for olfactory training using the developed device, which mainly consist of four steps. Firstly, the users select the category of liquid odorants, which are stored in different bottles in advance. The nine selected bottles should be numbered in sequence for distinction. Both the duration time for each odor and the interval for each subtest should be set in this step. During the training, the users do not need to continuously open and close the sniffing sticks for the participants. They only need to press the buttons of different numbers, and then the odor in that bottle will be released into the gas vessel.

**Figure 9:**
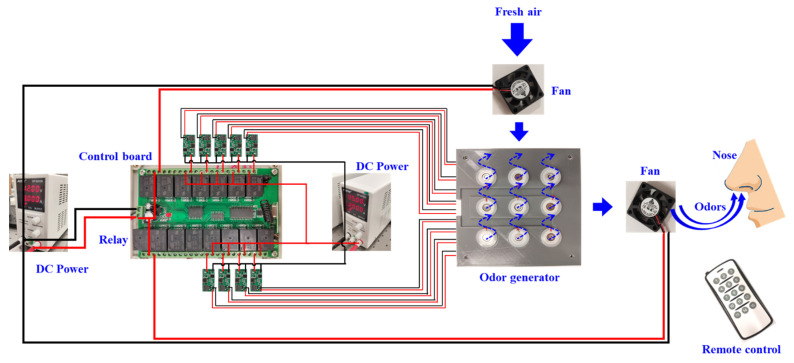
Control board of Flexible Odor-Releasing Device.

Secondly, these setting signals will be transmitted to the controller through a Bluetooth module. The control system for the device includes a power supply, a control board, and a Bluetooth module. Figure 9 demonstrates the scheme diagram of the control system. The atomizer needs to operate on 5 V power, but the operation of the fans and the control board requires 12 V power. Therefore, two DC power supplies were employed for the device. The users can replace them with batteries for portability of the application. There are 15 individual relay outputs on the control board, which are sufficient for the operation control of 9 bottles and 2 fans. These relays have two operating modes. In the manual mode, the relay only works when the users continue to press the button. The mode is applicable to random control training, in which the release time and interval need not be previously set before training. The examiner can flexibly provide different odors to the participants. In the auto mode, the users just need to click the button to start the training and click one more to stop it; this mode is designed for programmed training. In practice, users can conveniently select the mode, based on their specific requirements. The Bluetooth module is designed for the communication between the board and the remote control, which allows for the isolation of the participants from the examiners, eliminating unnecessary factors influencing the training results. Examiners only need to begin the training by pressing the buttons on the remote control. Thirdly, the odor generation system will receive the signals from the controller and be launched to produce the odors. The two fans should be first activated to clean up the gases in the vessel before each subtest. Then, the ultrasonic atomizer starts to produce odors from the scent bottles into the vessel. The odors will then be further mixed with the fresh air and be conveyed to the participant’s nose by the two fans. Finally, the odors will enter the olfactory cleft to help improve the function of the olfactory neurons of the participant.

In summary, compared with the sniffing sticks, this novel device can help both the examiners and the participants conveniently perform olfactory training. The main advantages are summarized as follows:

- Low-cost: the components of the hardware are all cheap open parts, making the training affordable and beneficial to more people;
- Customizable: the scent bottles are not constant; the odorants and their concentrations in the bottles, as well as the different training periods, can be specially customized according to the different symptoms and needs of various participants;
- Semi-automatic: to provide training, the examiners need only to press some buttons, avoiding handling various sticks. Participants can even conduct the training by themselves, based on the examiners’ instructions;
- Flexible: the overall dimension and weight of the device are as small as those of the sniffing sticks. Therefore, olfactory training based on this method can be flexibly conducted, both for the examiners and the participants, in more scenarios. Moreover, the odors can be freely combined and simultaneously released to increase the flexibility of the training.

## 3. Experiments

**Figure 10:**
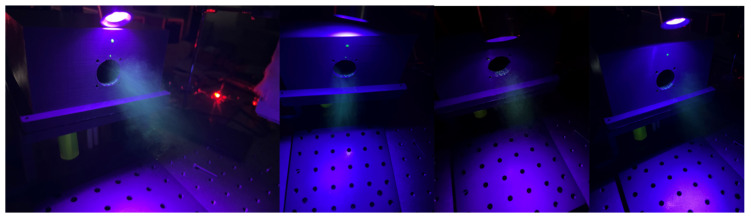
Practical results of the odor generation with different flow rates.

In order to further assess the performance of the developed device, experiments on odor generation were also conducted. Odors are commonly colorless gases. It is hard for the naked eyes to distinguish the odor particles from the ultrasonic atomizer. Therefore, the method of fluorescence measurements was employed in this paper to assess the performance of odor generation. In a scent bottle, 0.05 mL odorless fluorescent was added to the 15 mL odorant liquid and was well mixed with the liquid. Then, both the fluorescent and the odorant were atomized and released into the gas vessel. The fresh air conveyed by the two fans carried them out of the vessel. A purple light was placed near the exit position, and the gases exhausted from the vessel could be easily spotted under the light. Before the experiment, the odorants and device were left standing for 4 h to check the leakage of the atomizer, and the results showed that scarcely any leakage spots were found on the surface of the atomizer. That is, for one common training period (usually less than 1 h), the leakage of the odors from the atomizer owing to their volatility is nearly negligible. Figure 10 shows the captured images (resolution 3024 × 4032, ISO 800, aperture 1.5, and exposure time 1/30 s) of the odors with different flow rates during the experiments. The flow rates of the gases were controlled by adjusting the rotation speed of the two fans. As shown, the brightness of the exhausted gases from the exit becomes weaker with an increasing flow rate. The generation rate of odor particles by the atomizer was constant, but the flow rate of the fresh air increased with higher rotation speeds of the fans. As a result, the concentration of the odor particles in the mixing gases decreases and their brightness dims. In other words, the device has realized the function of providing odors with different concentrations to the participants, as do the sniffing sticks, but the odors were delivered by controlling the flow rate of the fresh air using the two fans.

**Figure 11:**
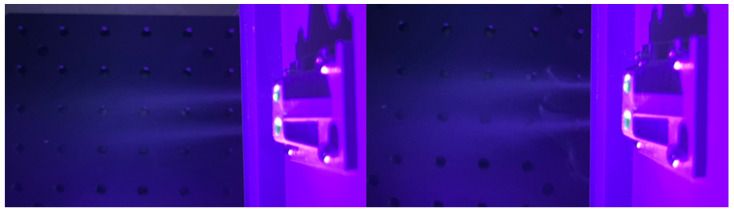
Odor flows through an artificial nose structure.

It is well known that the nose is comprised of two nostrils. In order to provide a better training effect, a structure with two through-holes of 5 mm each was designed at the exit of the gas vessel to help the participants efficiently sniff the odors. Gas flows via the structure were also captured, as shown in Figure 11 (resolution 4032 × 3024, ISO 1600, aperture 1.5, and exposure time 1/15 s). Compared with the results in Figure 10, it can be clearly seen that the distribution of the flow turned from divergence to concentration with this structure. The new odor flow is comprised of a long, thin structure. With this flow, the participants’ noses will be accurately exposed to the odors, and will scarcely be affected by the surrounding environment; the odors can precisely enter the olfactory cleft of the participant. Moreover, the device can also realize the contact-free training between the device and the participants with such a straightforward gas flow, which can protect the device from possible pollution.

## 4. Conclusions and Future Works

In this study, a novel device, which is low-cost, customizable, semi-automatic, and flexible, was developed for olfactory training based on 3D printing. This device can help the participants conveniently and accurately conduct their olfactory training. The device mainly contains three parts, i.e., a framework, an odor generator, and a controller with power supplies. The framework is responsible for the support and stability of all the components in the device. The odor generator is based on an ultrasonic atomizer, which has been proven effective by the experimental results in this study. The atomizer has a very simple working principle, but contains power functions for the application of odor generation. The controller provides power to the electronic components and controls the whole workflow of the device. In the training, the values of overall training time, sniffing time, and the interval of each subtest can be predefined for the device through the controller. Then, the examiner only needs to press a few buttons on the remote controller to launch the tests, and the examiner does not need to have contact with the participants. Based on detachable scent bottles, the odorants and their concentrations can be specifically selected according to the different requirements of the participants. Experiments regarding odor generation were further conducted to evaluate the performance of the developed device, and the results have proven the practicability of olfactory training based on this new device.

In future works, the quality of the device will be further improved to make it more easily reproducible. The criteria for the selection of scent bottles and odorants are critical for the users. Therefore, these should be established as soon as possible. Although many improvements can still be made to the developed device, it nevertheless shows huge potential in the field of olfactory training, and it will be proven to be a powerful tool for the treatment of olfactory dysfunction.
